# Effect of HIV infection on the expression and the activity of the proteasome in primary CD4 T cells

**DOI:** 10.1186/1742-4690-9-S2-P262

**Published:** 2012-09-13

**Authors:** J Boucau, CS Carlin, J Madouasse, M Shimada, S Le Gall

**Affiliations:** 1 Ragon Institute of MGH, MIT and Harvard, Charlestown, MA, USA

## Background

HIV-specific CD8 T cells responses rely on the recognition of peptide-MHC-I complexes by cognate T cell receptors. HIV-derived MHC-I epitopes result from the degradation of viral proteins by the cellular processing machinery including proteasomes and aminopeptidases. Interferon gamma changes proteasome composition and peptidase activities. We hypothesize that HIV infection might affect the expression or activities of the antigen processing machinery, either through a direct effect of the virus or indirectly through cellular activation or from the release of cytokines by surrounding infected cells.

## Methods

Primary CD4 T cells were selected from peripheral blood mononuclear cells of HIV seronegative individuals, and then activated with phytohemagglutinin or anti-CD3/CD28 beads prior to infection with HIV NL4-3. Cell activation was assessed by flow cytometry. The expression of proteasomal subunits was quantified by western blot at 4 days post activation or at 12 to 18 days post infection. Peptidase activities of CD4 T cells were characterized using a fluorescence-based assay.

## Results

In CD4 T cells isolated from 15 donors, the expression of 2 proteasome catalytic, 2 non-catalytic and 2 19S lid subunits significantly decreased with increasing percentage of HIV-infected cells (p<0.05). Accordingly, the chymotryptic activity of the proteasome decreased upon HIV-infection (p=0.027). After PHA activation, peptidase activities increased and the fold change in aminopeptidase and caspase-like peptidase activities between PHA-activated and non-activated samples correlated with the percentage of CD25-positive cells (p<0.01). The in vitro degradation of HIV peptide in extracts from T cells analyzed by mass spectrometry shows differences in epitope production between non-activated and PHA-activated cells.

## Conclusion

Both HIV infection and cellular activation affect the expression and peptidase activities of the antigen processing machinery of primary CD4 T cells which may impact the presentation of epitopes by both uninfected and infected cells during HIV infection.

This project was supported by NIH-NIAID grants R01 A1084106 and P01 A1074415.

